# Pigmentation in *Drosophila melanogaster* reaches its maximum in Ethiopia and correlates most strongly with ultra-violet radiation in sub-Saharan Africa

**DOI:** 10.1186/s12862-014-0179-y

**Published:** 2014-08-13

**Authors:** Héloïse Bastide, Amir Yassin, Evan J Johanning, John E Pool

**Affiliations:** 1 Laboratory of Genetics, University of Wisconsin-Madison, Madison, WI, USA

**Keywords:** Melanism, Drosophila, Thoracic trident, Bogert’s rule, Gloger's rule, Adaptation, UV resistance

## Abstract

**Background:**

Pigmentation has a long history of investigation in evolutionary biology. In *Drosophila melanogaster*, latitudinal and altitudinal clines have been found but their underlying causes remain unclear. Moreover, most studies were conducted on cosmopolitan populations which have a relatively low level of genetic structure and diversity compared to sub-Saharan African populations. We investigated: 1) the correlation between pigmentation traits within and between the thorax and the fourth abdominal segment, and 2) their associations with different geographical and ecological variables, using 710 lines belonging to 30 sub-Saharan and cosmopolitan populations.

**Results:**

Pigmentation clines substantially differed between sub-Saharan and cosmopolitan populations. While positive correlations with latitude have previously been described in Europe, India and Australia, in agreement with Bogert's rule or the thermal melanism hypothesis, we found a significant negative correlation in Africa. This correlation persisted even after correction for altitude, which in its turn showed a positive correlation with pigmentation independently from latitude. More importantly, we found that thoracic pigmentation reaches its maximal values in this species in high-altitude populations of Ethiopia (1,600-3,100 m). Ethiopian flies have a diffuse wide thoracic trident making the mesonotum and the head almost black, a phenotype that is absent from all other sub-Saharan or cosmopolitan populations including high-altitude flies from Peru (~3,400 m). Ecological analyses indicated that the variable most predictive of pigmentation in Africa, especially for the thorax, was ultra-violet (UV) intensity, consistent with the so-called Gloger's rule invoking a role of melanin in UV protection.

**Conclusion:**

Our data suggest that different environmental factors may shape clinal variation in tropical and temperate regions, and may lead to the evolution of different degrees of melanism in different high altitude populations in the tropics.

## Background

Melanism, *i.e.* the presence of dark forms within a species [1], has a long history in evolutionary biology. Two lines of research have been undertaken: dissecting genetic loci that contribute to the development of melanic forms, and exploring the external ecological and historical factors that maintain or drive the evolution of melanism. The first line of research has led to the identification of sets of orthologous genes responsible for the synthesis of tyrosine-derived melanin in different animal lineages. Most notable is the recurrent evolution of melanism due to independent mutations in *MC1R* gene in vertebrate species [2],[3]. The second line has usually aimed to associate melanic polymorphism with different environmental clines, such as the industrial melanism of the peppered moth [1],[4], and Gloger's rule in endotherms and Bogert's rule in ectotherms stating that pigmentation should decrease and increase with latitude, respectively [5].

Most of our knowledge about the developmental basis of melanin synthesis in insects draws from studies on *Drosophila*. Regulatory mutations in the *yellow*, *ebony*, *tan* and *bric-à-brac* genes have driven the evolution of melanism within and between different species [6]–[10]. In *Drosophila melanogaster*, two pigmentation traits have traditionally been investigated in natural populations: the thorax (mesonotum) which when darkly pigmented forms a black trident [11], and the abdomen, measured either as stripe width [12] or background pigmentation [13] on different segments. Geographical clines of the thoracic trident were shown relative to the latitude in Europe [14], India [15] and Australia [16], and to altitude in India [15],[17]. In sub-Saharan Africa, no latitudinal cline for thoracic pigmentation was found [14]. Clines were also found in *D. melanogaster* abdominal pigmentation relative to latitude/altitude in India [18]–[20] and altitude in sub-Saharan Africa [13]. Geographical clines of abdominal pigmentation also occur in other species relative to latitude (*D. dunni* subgroup, [21]; *D. simulans*, [22]), altitude (*D. immigrans*, [23]; *D. kikkawai*, [24]; *D. yakuba*, [25]), longitude/aridity (*D. americana*, [26]), and forest density (*D. polymorpha*, [27]).

Sub-Saharan Africa harbors the ancestral range of *D. melanogaster*, while its cosmopolitan populations are thought to derive from a single 'out-of-Africa' event [28]–[30]. Moreover, the topography of Africa is a mosaic of lowlands and highlands that does not follow a latitudinal gradient. Such a rich genetic and topographic diversity is ideal for the investigation of the environmental factors that may contribute to the development of pigmentation clines. Several hypotheses have been proposed to explain clinal variation of *Drosophila* pigmentation (reviewed in [31]). The most invoked one, known as the 'thermal budget hypothesis' [14],[18] or 'thermal melanism' [32], states that dark cuticle is adaptive in cold habitats for its higher absorbance of solar radiation. Other hypotheses aimed to relate differences in pigmentation to desiccation tolerance [19],[20],[27],[33] or ultra-violet (UV) resistance [25],[34]–[36]. Although crypsis with substrate/soil color was invoked as one of the best explanations for industrial melanism [1] and clinal variation in rodents [37], it has rarely been suggested for drosophilids [27],[38]. Other biotic factors are known to be affected by pigmentation in *Drosophila*, such as immunity against infection [39] and sexual selection [40], but clinal variations due to these factors have not yet been investigated to our knowledge. these factors seem less likely to drive clinal variations. Although the different hypotheses are not mutually exclusive, it remains difficult to understand which is the major force or forces driving global or local clinal variation in *D. melanogaster*.

In this paper, we aimed at simultaneously analyzing latitudinal and altitudinal clines of both abdominal and thoracic pigmentations in sub-Saharan *D. melanogaster* populations. We are not aware of any previous study that simultaneously investigated the two characters in the same set of populations, but a recent experiment indicated a substantial degree of correlated response to artificial selection [41]. We thus investigated the correlation between different traits of pigmentation within and between the thorax and the fourth abdominal segment (A4), and investigated their correlations with different ecological variables. The recent availability of large GIS databases of climatological and geological data and computational tools now enables a better dissection of these environmental causes [5]. Previous studies emphasized the roles of temperature and aridity in the development of pigmentation clines in *D. melanogaster*, but our results indicate that the neglected role of UV resistance may be more relevant, at least in tropical Africa.

## Methods

### Fly populations

**Figure 1:**
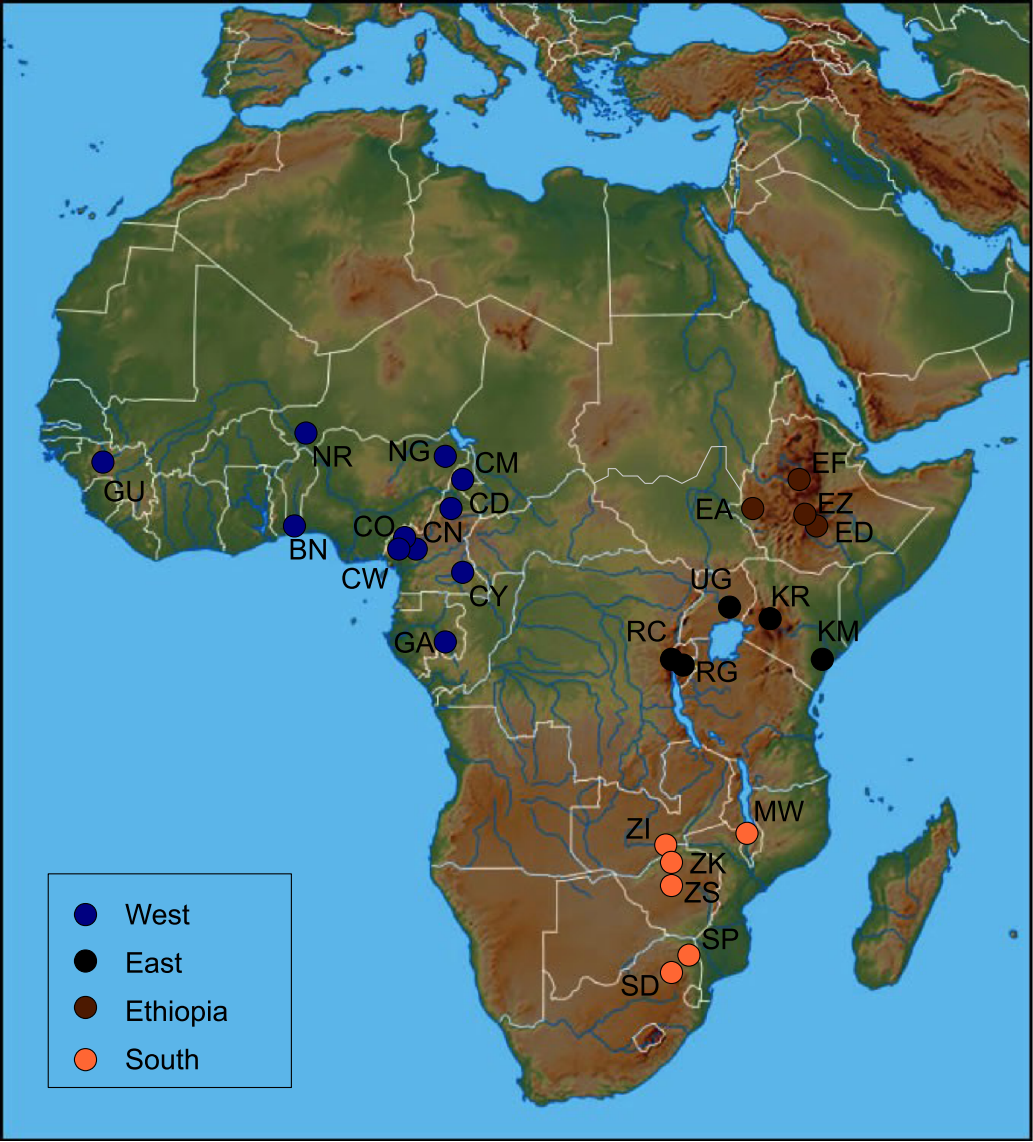
***Drosophila melanogaster*sub-Saharan populations used in this study.** Colors refer to populations division into four subclans according to their genetic structure.

Table 1 shows the list of the 30 geographical populations that we used in our study. These populations were classified into two major clans: sub-Saharan and cosmopolitan (26 and 4 populations, respectively). The sub-Saharan clan was further divided into four subclans according to their population genetic structure revealed from recent genomic data [30]: west, east, ethiopian and south African subclans (11, 5, 4 and 6 populations, respectively; Figure 1).

**Table 1:** Populations sampled in this study

**Population**	**Symbol**	**Latitude**	**Longitude**	**Altitude**	**Date**	**Data set**	***N***
**Sub-Saharan Africa**							
**West**							
Dondé, Guinea	GU	10.70	−12.25	801	06/2004	I	21
Cotonou, Benin	BN	6.35	2.43	52	05/2004	I	8
Maiduguri, Nigeria	NG	11.85	13.16	295	09/2004	I	9
Kareygorou, Niger	NR	13.55	2.03	195	12/2004	I	20
Yokadouma, Cameroon	CY	3.52	15.05	561	04/2004	I	8
Mbalang-Djalingo, Cameroon	CD	7.32	13.73	1213	03/2004	I, D	20, 6
Maroua, Cameroon	CM	10.60	14.32	385	03/2004	I	22
Nkouondja, Cameroon	CN	5.50	10.68	1121	04/2004	I, D	24, 6
Mbengwi, Cameroon	CW	6.02	10.00	1274	04/2004	I, D	6, 4
Oku, Cameroon	CO	6.25	10.43	2169	04/2004	I, D, M	9, 5, 10
Franceville, Gabon	GA	−1.65	13.60	332	03/2002	I	19
**East**							
Namulonge, Uganda	UG	0.53	32.60	1134	04/2005	I, D, M	20,21,17
Cyangugu, Rwanda	RC	−2.29	28.55	1602	12/2008	D	14
Gikongoro, Rwanda	RG	−2.49	28.92	1927	12/2008	D, M	25, 25
Marigat, Kenya	KR	0.47	35.98	1062	01/2009	D	25
Malindi, Kenya	KM	−1.43	40.03	78	01/2009	D	21
**Ethiopia**							
Gambella, Ethiopia	EA	8.25	34.59	525	12/2011	M	6
Ziway, Ethiopia	EZ	7.93	38.72	1642	12/2008	D	25
Dodola, Ethiopia	ED	6.98	39.18	2492	12/2008	D	7
Fiche, Ethiopia	EF	9.81	38.63	3070	12/2011	M	44
**South**							
Mwanza, Malawi	MW	−15.62	34.52	618	07/2001	I, D	13, 11
Siavonga, Zambia	ZI	−16.54	28.72	530	07/2010	M	51
Lake Kariba, Zimbabwe	ZK	−16.52	28.80	619	05/1994	I	16
Sengwa, Zimbabwe	ZS	−18.16	28.22	865	09/1990	I	13
Phalaborwa, South Africa	SP	−23.94	31.14	375	07/2010	M	6
Dullstroom, South Africa	SD	−25.42	30.10	2000	12/2011	M	49
**Cosmopolitan**							
**Palearctic**							
Cairo, Egypt	EG	30.10	31.32	25	01/2011	M	9
Lyon, France	FR	45.77	4.86	175	07/2010	M	71
**Neotropical**							
Puerto Maldonado, Peru	PM	−12.60	−69.19	200	04/2013	M	14
Cusco, Peru	PC	−13.52	−71.97	3400	04/2013	M	10

*N* = number of lines.

Populations were also divided into three sets of data according to the location and date of pigmentation scoring: *I* (Cornell University, Ithaca 2005), *D* (University of California, Davis 2009) and *M* (University of Wisconsin, Madison 2013). The *I* data set includes populations whose abdominal pigmentation scores have already been published by Pool and Aquadro [13]. Among the 19 sub-Saharan populations studied by these authors, we excluded three populations from Eritrea, Kenya and South Africa due to elevated genomic evidence of recent admixture from cosmopolitan strains [30]. The *I* and *D* data sets consisted of 228 and 170 isofemale lines, whereas *M* consisted of 312 lines that were inbred for eight generations (Table 1).

### Scoring pigmentation

For each line, five males and ten females were maintained at 20°C and ~75% humidity on standard *Drosophila* medium (containing molasses, corn meal, yeast, agar, and antimicrobial agents). One, 3–5 day old female from the progeny per line was photographed using an Amscope SM-4TZZ-144A dissection microscope under CO_2_ anesthesia. For the *I* and *D* data sets flies were photographed on lateral view, but for *M* both lateral and dorsal views were taken. Photos were then analyzed using the ImageJ software package [42].

**Figure 2:**
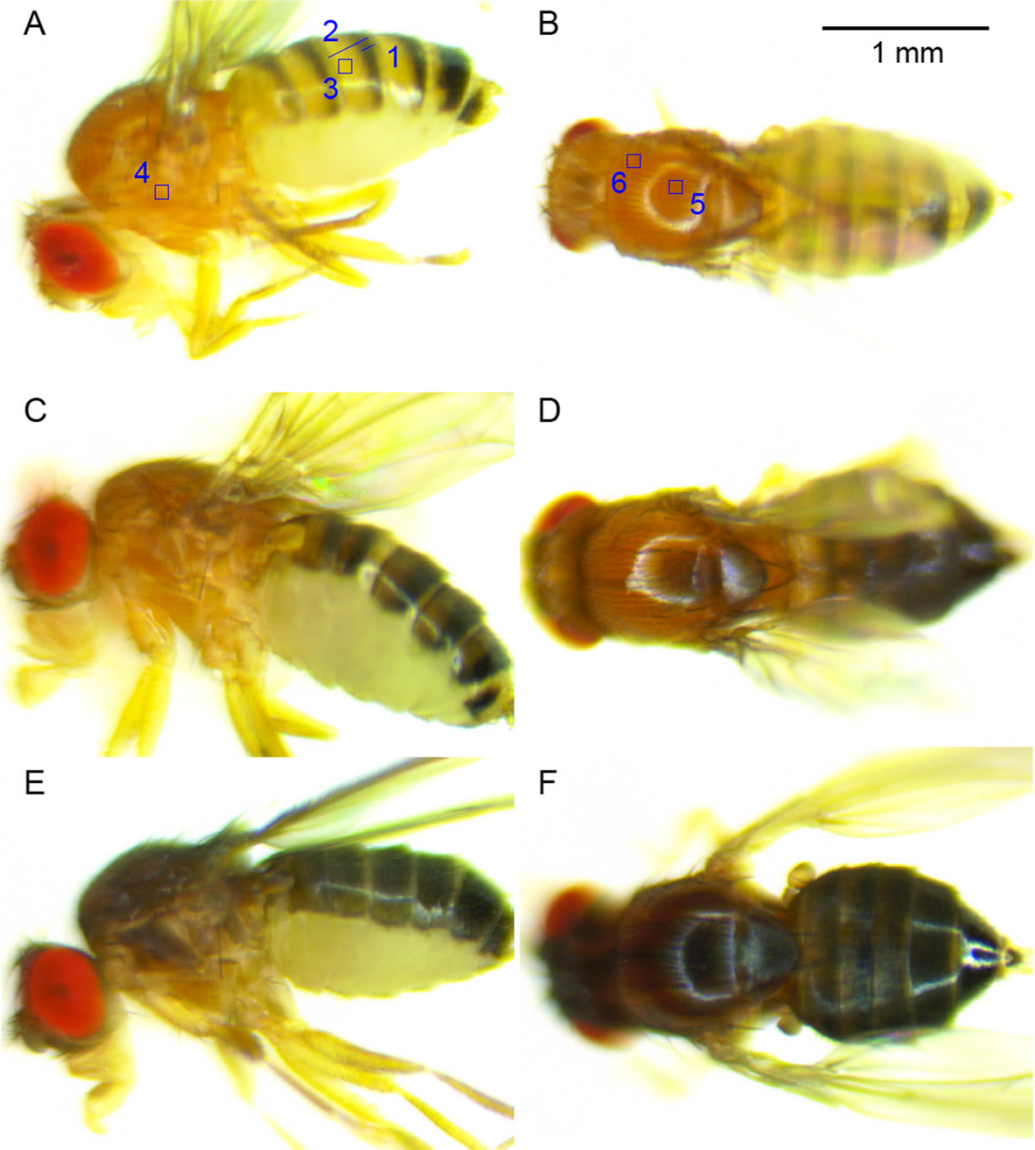
**Natural variation of thoracic and abdominal pigmentation between three sub-Saharan populations of*Drosophila melanogaster*: (A-B) typically pigmented flies from Siavonga, Zambia, (C-D) moderately melanic flies from Nkouondja, Cameroon, and (E-F) intensely melanic flies from Dodola, Ethiopia.** Pigmentation traits measured in this study on **(A)** lateral and **(B)** dorsal views: 1 = A4 black stripe width, 2 = A4 total tergite width, 3 = A4 background, 4 = mesopleural (MPL), 5 = presutural, 6 = humeral.

We scored different pigmentation traits on two cuticular structures: the fourth abdominal segment and the (thoracic) mesonotum (Figure 2). For abdominal pigmentation two traits were measured: pigmentation intensity near the anterior margin (A4 background), measured in greyscale % as in Pool and Aquadro [13], and width of the posterior black stripe (A4 stripe) as in David et al. [12]. For the latter trait, we divided the width in pixels of the black stripe by the whole width in pixels of A4, rather than visually estimating the ratio. Both traits were measured approximately half-way between the dorsal midline and the dorsal-ventral (tergite-sternite) boundary. A4 stripe was measured for flies from the *M* data set only. For the thorax, three pigmentation intensity traits were scored in greyscale %: on the latero-anterior margin of the scutum near the humerus, on the posterior margin of the scutum near the scuto-scutellar suture, and on the mesopleuron on the katepisternal sclerite above the level of the two large katerpisternal bristles (hereafter MPL). In addition, we scored the thoracic trident according to the four phenotypic classes proposed by David et al. [14]: 0 = no trident, 1 = faint trident, 2 = clearly marked trident, and 4 = dark trident. Because humeral, presutural and trident pigmentation traits need to be scored from a dorsal perspective they were only scored for flies from the *M* data set, whereas MPL was scored for all data sets.

Exposure time, zoom width and illumination level using an Amscope adaptor for LED lamp at maximum lighting were kept constant within each data set but they differed due to different laboratory equipments between the three sets. Flies were microphotographed on a fixed region of the CO_2_ pad, to minimize background effects. Since some strains were measured in different sets (Table 1), their A4 background means were used to scale greyscale measurements of *D* and *M* relative to *I* using a linear model. To scale *D* relative to *I*, three populations were used (MW = 53.6 and 62.6, UG = 68.2 and 74.0, and RG = 61.4 and 72.1, with scores indicating population means for measurements made in *D* and *I*, respectively). This led to a relationship of *x*_*I*_ = 21.23 + 0.79 *x*_*D*_ (*R*^*2*^ = 0.89), with *x*_*D*_ being the mean of measurements scored in Davis and *x*_*I*_ the scaled measurement relative to Ithaca. To scale *M* relative to *I*, three data points were also used, corresponding to CO, UG and ZK/ZI, with the latter being two populations from Zimbabwe and Zambia, respectively, that are less than 10 km apart and share similar habitats. The pigmentation scores (CO = 62.0 and 72.1, UG = 59.4 and 74.0, and ZI/ZK = 41.8 and 61.8, with measurements indicating means for *M* and *I*, respectively) led to a relationship of *x*_*I*_ = 37.92 + 0.58 *x*_*M*_ (*R*^*2*^ = 0.93), with *x*_*M*_ being the mean of measurements scored in Madison and *x*_*I*_ the scaled measurement relative to Ithaca. These rescalings are unlikely to perfectly account for the differences between data sets, which may add noise to some analyses. However, we do not expect any strong bias in terms of environmental correlations, and we find no significant effect of data set on rescaled A4 and MPL pigmentation scores for rescaled A4 and MPL pigmentation scores, respectively: Kruskal-Wallis chi-squared = 4.46 (*P* = 0.1073) and 4.19 (*P* = 0.1233).

### Statistical analyses

Three main statistical analyses were conducted in this paper. First, we evaluated the levels of phenotypic correlations between the five pigmentation traits scored within two sub-Saharan populations representing phenotypic extremes of the *M* data set. To correct for multiple correlation tests, we estimated the false discovery rate (FDR) for a given *P* value, *i.e.* a *q* value, and significance levels were considered at *q* < 0.05, *i.e.* a 5% FDR cutoff.

Second, we simultaneously estimated latitudinal and altitudinal clines for the two measurements of abdominal and thoracic pigmentations (A4 background and MPL) scored on the three populations data sets, using a multiple linear regression model as in Munjal et al. [15]:

(1) $$y=a+b_{1\;}\mathrm{lat}.+b_{2}\;\mathrm{alt}.+\varepsilon$$

where *y* is the pigmentation score mean of a population, *a* is the intercept, *b*_*1*_ and *b*_*2*_ are the slopes (or the clines) of the absolute latitude and altitude values, and *ε* is the residual. Analyses were conducted for sub-Saharan populations only. For comparative purposes, we also independently estimated the zeroth-ordered correlation coefficient (*r*_0_) between each pigmentation trait, latitude and altitude, as well as the semi-partial correlation coefficient (*r*_s_) of each pigmentation trait with the latitude or the altitude after controlling for the effect of the other geographical variable on pigmentation.

Third, we analyzed the correlation between A4 background and MPL and different ecological and historical factors that may affect pigmentation evolution. In order to determine the environmental factors that may explain each of the clines, we obtained GIS meteorological and geological data for each population. Meteorological data averaged over 22 years (from 1983 to 2005) were extracted as annual averages from the NASA Surface meteorology and Solar Energy: Global Data Sets website (www.eosweb.larc.nasa.gov). These included five radiation sets (namely, insolation incident, diffuse radiation, direct normal radiation and latitude tilt radiation, each measured in kWh/m^2^/day) and seven climatic sets (namely, average, minimum and maximum air temperatures and earth temperature measured in °C, relative humidity in %, atmospheric pressure in kPa, and wind speed at 50 m above ground in m/s). We also included annual average values of UV index from the Tropospheric Emission Monitoring Internet Service (www.temis.nl) in units of 25 mW/m^2^. Geological data of soil sand, silt and clay contents at <2 μm in % were obtained from the Africa Soil Information Service (www.ciesin.columbia.edu/afsis). We estimated the coefficients of correlation between pigmentation traits and each variable, and then chose for each ecological category that has previously been hypothesized to affect pigmentation (*i*.e. UV protection, thermal budget, desiccation and crypsis) a single variable showing the strongest correlation with pigmentation. In addition we included two principal component scores, namely PC1 and PC2, from Pool et al. [30] reflecting subclan differentiation to control for population structure and historical effects. To evaluate the relative importance of each of these ecological or historical factors, we used an information-theoretic approach that ranks a number of linear model-based hypotheses according to evidence ratios and posterior probabilities [43]. We conducted this analysis first using each factor as a separate model, and then using each factor and combination of two factors as models. For each model, the sample-size corrected Akaike information criterion (AICc) was estimated, and models were ranked on the basis of increasing AICc and evaluated in respect to the descending probabilities (*w*).

All statistical analyses were performed using the R software package (www.r-project.org). Semi-partial and second-ordered partial correlation analyses were conducted using the ppcor 1.0 package [44] in R. False Discovery Rate (FDR) control [45] was applied for multiple tests of phenotypic correlations between traits using the LBE 1.22 software package [46] in R. For model set evaluation, we modified the R code provided by Correa and Hendry [47] to fit our data.

## Results

### Correlation between pigmentation traits

The lowland, light ZI population from Zambia (occupying the suggested ancestral range of this species [30]) and the highland, dark EF population from Ethiopia represented the two pigmentation extremes of the *M* data set (Figure 2). In both populations, correlations between pigmentation traits within each of the two segments were positive and significant (Table 2). However, the two populations differed in the sign and significance of the correlations between the segments. In Zambia, no significant correlation was found between an abdominal pigmentation trait and a thoracic trait and some coefficients had negative values. In Ethiopia, all coefficients were significantly positive after FDR correction for multiple comparisons, possibly indicating a correlated genetic basis of abdominal and thoracic melanism in this population. The correlation between MPL, *i.e.* pigmentation on the pleura, and the trident, indicates that this trait should be moderately predictive of trident polymorphism in populations of the *I* and *D* data sets, for which no photomicrographs of the dorsal view were available. Interestingly, humeral pigmentation at the anterior portion of the thorax was only correlated with presutural pigmentation at the posterior portion of the scutum. Variation in thoracic background color is therefore not necessarily linked to either thoracic trident or abdominal pigmentation traits.

**Table 2:** Correlation coefficients between different pigmentation traits within a lowland population (ZI, below the diagonal) and a highland population (EF, above the diagonal)

	**A4 background**	**A4 stripe**	**Humeral**	**MPL**	**Presutural**	**Trident**
A4 background		0.332**	0.525***	0.646***	0.673***	0.492***
A4 stripe	0.344*		0.107	0.202*	0.260*	0.192*
Humeral	−0.004	−0.245		0.678***	0.567***	0.374**
MPL	0.232	0.198	0.210		0.638***	0.270*
Presutural	0.220	−0.128	0.728***	0.546*		0.661***
Trident	0.106	0.024	0.282	0.458**	0.823***	

A4 = 4^th^ abdominal segment, MPL = mesopleuron. Significance levels (*q* values) are after FDR correction for multiple comparisons.

*q < 0.05; **q < 0.01; ***q < 0.001.

### A new phenotype of thoracic pigmentation in Ethiopia

**Figure 3:**
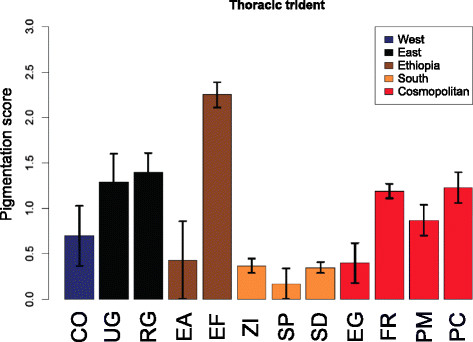
**Average thoracic trident pigmentation scores in*D. melanogaster*populations of the*M*data set.** Error bars indicate standard errors of the means.

A uniquely melanic phenotypic class which goes beyond the scale defined by David et al. [14] was found and scored for some lines from the high-altitude Ethiopian population EF. These phenotypes have a diffuse wide trident on the thorax making the mesonotum (and the head) appearing completely black (Figure 2). They were scored as a fifth class (value = 4), a phenotype that was absent in any previously examined sub-Saharan or cosmopolitan strain of *D. melanogaster* or its close relative *D. simulans* (J. R. David, *pers. comm.*). Consequently, the EF mean of thoracic trident was nearly twice that of other high latitude or altitude populations (e.g., France, Peru, Cameroon, Uganda and Rwanda) (Figure 3). The phenotype was also found in another high altitude Ethiopian population (ED), but it was absent from the low-altitude Ethiopian population EA.

### Geographical differentiation of pigmentation traits

**Figure 4:**
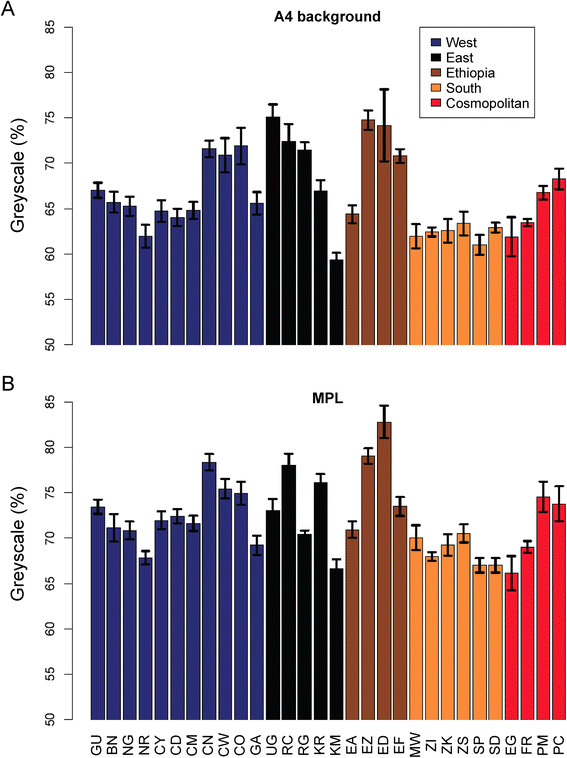
Population means of (A) A4 and (B) MPL pigmentation scores of all data sets.

A4 background and MPL pigmentation variation between populations in all data sets after calibration with the scale of the *I* data set showed similar geographical patterns between genetic clans (ANOVA's *F* = 3.09 (*P* = 0.0134) vs. 3.22 (*P* = 0.0292), respectively; Figure 4). The two traits did not differ, however, in their variance, with the coefficient of variation being 6.74% for A4 background and 5.63% for MPL (*F*-test = 1.43; *P* = 0.83, [48]). Tukey's pairwise test showed a single significant pigmentation difference between Ethiopian and south African populations (Table 3; *P =* 0.0127 and 0.0152 for A4 background and MPL, respectively). Remarkably, the uniquely melanic thoracic phenotype of high-altitude Ethiopian populations found at altitudes ranging between 1,600 and 3,100 m was not found in any other high-altitude sub-Saharan or even cosmopolitan population, including the population from Cusco, Peru (3,400 m). Melanism from Ethiopian highland strains thus appears to greatly exceed the pigmentation of any previously described *D. melanogaster* populations.

**Table 3:** Tukey’s ad hoc comparisons of A4 background (below diagonal) and MPL (above diagonal) between genetic subclans

	**West**	**East**	**Ethiopia**	**South**	**Cosmopolitan**
West		0.38	4.09	3.85	1.60
East	2.37		3.71	4.23	1.97
Ethiopia	4.36	1.99		7.94*	5.69
South	4.30	6.67	8.66*		2.25
Cosmopolitan	1.57	3.94	5.93	2.73	

*P < 0.05; **P < 0.01; ***P < 0.001.

### Opposite latitudinal clines between sub-Saharan and cosmopolitan populations

**Figure 5:**
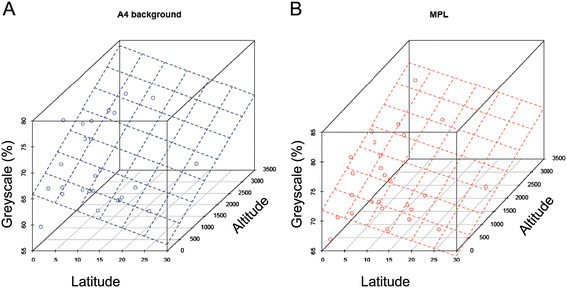
**Negative latitudinal and positive altitudinal clines of population means of (A) A4 background and (B) MPL pigmentation scores in sub-Saharan Africa.** The planes indicate the slopes from the multiple regression analyses.

A4 background and MPL pigmentation in sub-Saharan Africa were significantly correlated with both latitude and altitude (Table 4; Figure 5). This situation is similar to what has previously been found on the Indian subcontinent [15],[19]. Although in both cases pigmentations' slopes were positive for altitude, *i.e.* populations inhabiting high altitudes are darker, a negative slope was found for latitude in sub-Saharan Africa, whereas the latitudinal slope was positive in India. This difference is most likely due to the topographic nature of the Indian subcontinent where both latitudes and altitudes covary, *i.e.* elevations increase northward toward the Himalayan plateau. In Africa, latitudes and altitudes do not covary (*r* = −0.12, *P* = 0.56) and the pigmentation clines with each factor persist after controlling for the other factor (*r*_latitude_ = −0.64 (*P* = 6.7 × 10^−5^) and −0.52 (*P* = 0.004), *r*_altitude_ = 0.75 (*P* = 7.2 × 10^−8^) and 0.59 (*P* = 4.8 × 10^−4^), for A4 background and MPL, respectively).

**Table 4:** Multiple linear regressions of pigmentation traits on latitude and altitude in sub-Saharan Africa

**Variable**	**Intercept**	**Latitude**	**Altitude**
A4 background	65.866 ± 1.209***	−0.321 ± 0.081***	0.004 ± 0.001***
MPL	71.859 ± 1.336***	−0.257 ± 0.089**	0.003 ± 0.001**

*P < 0.05; **P < 0.01; ***P < 0.001.

### Pigmentation correlates most strongly with UV radiation in sub-Saharan Africa

**Figure 6:**
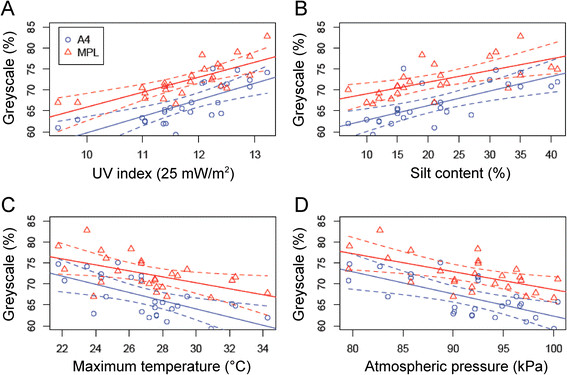
Linear regression of population means of A4 background (blue) and MPL (red) pigmentation scores on (A) ultra-violet (UV) index, (B) silt content, (C) average maximum temperature, and (D) atmospheric pressure, in sub-Saharan Africa.

Among the 18 environmental and historical factors considered in this study, pigmentation significantly correlated with 18 and 11 variables for A4 background and MPL, respectively (Table 5; Figure 6). For both traits UV index had the strongest correlations (*r* = 0.77 and 0.78 for A4 and MPL, respectively; Figure 6A). In order to evaluate the different ecological and historical hypotheses, we conducted a model rank analysis using five environmental and two population genetic structure factors. We found UV radiation to be the most explanatory factor for variation in sub-Saharan Africa, when each environmental factor is considered separately (g_01_ in Tables 6 and 7). Flies are darker at higher levels of UV index (slope = 3.95 ± 0.74 (*P =* 1.7 × 10^−5^) and 3.58 ± 0.62 (*P =* 6.6 × 10^−6^), for A4 background and MPL, respectively). Soil silt content (g_05_) was always the second most explanatory factor (slope = 0.35 ± 0.07 (*P =* 4.3 × 10^−5^) and 0.28 ± 0.07 (*P =* 3.1 × 10^−4^), for A4 background and MPL, respectively), but for both traits it was less likely than UV index (evidence ratio: *E*_g01,g05_ = 2.6 and 49.0, for A4 background and MPL, respectively). Similar results were obtained when combinations of traits were analyzed in a multivariate regression context (Tables 8 and 9). For MPL, the first four models, which weighted for ~75% of the variance among models, were models including UV index alone or in combinations (Table 8). For A4 background, the most explanatory model which weighted for 61%, did not include UV index. Instead this model was explained by correlations with temperature and the first principal component of genetic differentiation (slopes = −1.08 ± 0.17 (*P =* 1.97 × 10^−6^) and 27.08 ± 5.51 (*P =* 5.7 × 10^−5^), respectively), although the second and third most likely combinations did include UV. Thus, the unique melanic phenotype in Ethiopia may have evolved to protect against UV radiation, which reaches its maximum levels (>300 mW/m^2^) in the Ethiopian highlands. However, this hypothesis requires further study, as does the potential role of other ecological factors.

**Table 5:** Correlation of A4 background and MPL with different ecological and historical variables in sub-Saharan Africa

	**A4 background**	**MPL**
**Radiation**		
UV index	0.737***	0.760***
Insolation incident	−0.157*	−0.005
Diffuse radiation	0.411**	0.300
Direct normal radiation	−0.319*	−0.186
Latitude tilt radiation	−0.279*	−0.136
Clear sky radiation	0.324*	0.389*
**Temperature**		
Average temperature	−0.596***	−0.495**
Average minimum temperature	−0.485**	−0.413*
Average maximum temperature	−0.640***	−0.532**
Earth temperature	−0.665***	−0.501**
**Humidity**		
Relative humidity	0.414**	0.256
Atmospheric pressure	−0.658***	−0.607**
**Wind**		
Wind speed	−0.305*	−0.227
**Soil**		
Sand	−0.612***	−0.520**
Silt	0.714***	0.652**
Clay	0.178*	0.113
**Genetic structure**		
PC1	0.465**	0.491**
PC2	−0.323*	−0.376*

Significance levels after FDR correction.

*q < 0.05; **q < 0.01; ***q < 0.001.

**Table 6:** Univariate model set evaluation for A4 background

**Model**	**Formula**	***k***	**RSS**	**Adjusted** ***R***^***2***^	**AICc**	**Model likelihood**	***w***
g_01_	UV index	3	250.36	0.52	139.76	1.00	0.68
g_05_	Silt content	3	269.41	0.49	141.67	0.39	0.26
g_04_	Atmospheric pressure	3	311.23	0.41	145.42	0.06	0.04
g_02_	Mean max. temperature	3	323.71	0.39	146.44	0.04	0.02
g_06_	Genetic PC1	3	430.20	0.18	153.84	0.00	0.00
g_03_	Relative humidity	3	454.76	0.14	155.28	0.00	0.00
g_07_	Genetic PC2	3	491.51	0.07	157.30	0.00	0.00
g_08_	naïve	2	548.85	0.00	157.60	0.00	0.00

*k* = number of parameter estimates, RSS = residual sum of squares, AICc = Akaike information criterion adjusted for the sample size, *w* = conditional model probability (likelihood of model *i* divided by the sum of model likelihoods).

**Table 7:** Univariate model set evaluation for MPL

**Model**	**Formula**	***k***	**RSS**	**Adjusted** ***R***^***2***^	**AICc**	**Model likelihood**	**w**
g_01_	UV index	3	178.56	0.56	130.97	1.00	0.98
g_05_	Silt content	3	243.52	0.40	139.04	0.02	0.02
g_04_	Atmospheric pressure	3	267.16	0.34	141.45	0.01	0.01
g_02_	Average maximum temperature	3	303.60	0.25	144.77	0.00	0.00
g_06_	Genetic PC1	3	321.44	0.21	146.26	0.00	0.00
g_07_	Genetic PC2	3	363.57	0.11	149.46	0.00	0.00
g_08_	naïve	2	423.30	0.00	150.85	0.00	0.00
g_03_	Relative humidity	3	395.63	0.03	151.66	0.00	0.00

*k* = number of parameter estimates, RSS = residual sum of squares, AICc = Akaike information criterion adjusted for the sample size, *w* = conditional model probability (likelihood of model *i* divided by the sum of model likelihoods).

**Table 8:** Multivariate model set evaluation for A4

**Model**	**Formula**	***k***	**RSS**	**Adjusted** ***R***^***2***^	**AICc**	**Model likelihood**	**w**
g_17_	Temp + PC1	4	157.74	0.69	130.56	1.00	0.61
g_08_	UV + Temp	4	173.83	0.66	133.09	0.28	0.17
g_11_	UV + Silt	4	189.09	0.63	135.28	0.09	0.06
g_16_	Temp + Silt	4	195.87	0.61	136.19	0.06	0.04
g_23_	AtmP + Silt	4	196.90	0.61	136.33	0.06	0.03
g_24_	AtmP + PC1	4	199.13	0.61	136.62	0.05	0.03
g_10_	UV + AtmP	4	206.78	0.59	137.60	0.03	0.02
g_19_	RH + AtmP	4	210.71	0.58	138.09	0.02	0.01
g_01_	UV	3	250.36	0.52	139.76	0.01	0.01
g_09_	UV + RH	4	228.52	0.55	140.20	0.01	0.00
g_20_	RH + Silt	4	237.10	0.53	141.16	0.01	0.00
g_27_	All	9	112.68	0.71	141.16	0.00	0.00
g_05_	Silt	3	269.41	0.49	141.67	0.00	0.00
g_13_	UV + PC2	4	249.97	0.50	142.53	0.00	0.00
g_12_	UV + PC1	4	250.00	0.50	142.54	0.00	0.00
g_04_	AtmP	3	311.23	0.41	145.42	0.00	0.00
g_02_	Temp	3	323.71	0.39	146.44	0.00	0.00
g_25_	AtmP + PC2	4	297.46	0.41	147.06	0.00	0.00
g_15_	Temp + AtmP	4	298.47	0.41	147.14	0.00	0.00
g_14_	Temp + RH	4	305.03	0.40	147.71	0.00	0.00
g_18_	Temp + PC2	4	323.17	0.36	149.21	0.00	0.00
g_21_	RH + PC1	4	383.28	0.24	153.65	0.00	0.00
g_22_	RH + PC2	4	383.91	0.24	153.69	0.00	0.00
g_06_	PC1	3	430.20	0.18	153.84	0.00	0.00
g_26_	PC1 + PC2	4	390.20	0.23	154.11	0.00	0.00
g_03_	RH	3	454.76	0.14	155.28	0.00	0.00
g_07_	PC2	3	491.51	0.07	157.30	0.00	0.00
g_28_	naïve	2	548.85	0.00	157.60	0.00	0.00

UV = UV index, Temp = average maximum temperature, RH = relative humidity, AtmP = atmospheric pressure, PC1 and PC2 = genetic principal components, *k* = number of parameter estimates, RSS = residual sum of squares, AICc = Akaike information criterion adjusted for the sample size, *w* = conditional model probability (likelihood of model *i* divided by the sum of model likelihoods).

**Table 9:** Multivariate model set evaluation for MPL

**Model**	**Formula**	***k***	**RSS**	**Adjusted** ***R***^***2***^	**AICc**	**Model likelihood**	**w**
g_08_	UV + Temp	4	153.29	0.61	129.82	1.00	0.25
g_11_	UV + Silt	4	154.44	0.60	130.01	0.91	0.22
g_01_	UV	3	178.56	0.56	130.97	0.56	0.14
g_10_	UV + AtmP	4	161.08	0.59	131.11	0.52	0.13
g_17_	Temp + PC1	4	169.94	0.56	132.50	0.26	0.06
g_24_	AtmP + PC1	4	170.22	0.56	132.54	0.26	0.06
g_13_	UV + PC2	4	176.26	0.55	133.45	0.16	0.04
g_09_	UV + RH	4	178.27	0.54	133.75	0.14	0.03
g_12_	UV + PC1	4	178.50	0.54	133.78	0.14	0.03
g_23_	AtmP + Silt	4	194.80	0.50	136.05	0.04	0.01
g_16_	Temp + Silt	4	211.45	0.46	138.18	0.02	0.00
g_05_	Silt	3	243.52	0.40	139.04	0.01	0.00
g_19_	RH + AtmP	4	236.64	0.39	141.11	0.00	0.00
g_20_	RH + Silt	4	239.69	0.38	141.44	0.00	0.00
g_04_	AtmP	3	267.16	0.34	141.45	0.00	0.00
g_27_	All	9	116.06	0.62	141.93	0.00	0.00
g_25_	AtmP + PC2	4	266.41	0.32	144.19	0.00	0.00
g_15_	Temp + AtmP	4	266.84	0.31	144.23	0.00	0.00
g_02_	Temp	3	303.60	0.25	144.77	0.00	0.00
g_26_	PC1 + PC2	4	278.17	0.29	145.31	0.00	0.00
g_06_	PC1	3	321.44	0.21	146.26	0.00	0.00
g_18_	Temp + PC2	4	299.19	0.23	147.21	0.00	0.00
g_14_	Temp + RH	4	302.17	0.22	147.47	0.00	0.00
g_21_	RH + PC1	4	315.41	0.19	148.58	0.00	0.00
g_07_	PC2	3	363.57	0.11	149.46	0.00	0.00
g_22_	RH + PC2	4	328.39	0.16	149.63	0.00	0.00
g_28_	naïve	2	423.30	0.00	150.85	0.00	0.00
g_03_	RH	3	395.63	0.03	151.66	0.00	0.00

UV = UV index, Temp = average maximum temperature, RH = relative humidity, AtmP = atmospheric pressure, PC1 and PC2 = genetic principal components, *k* = number of parameter estimates, RSS = residual sum of squares, AICc = Akaike information criterion adjusted for the sample size, *w* = conditional model probability (likelihood of model *i* divided by the sum of model likelihoods).

## Discussion

### Pigmentation as a modular trait

Insect pigmentation can be regarded as a modular trait: its development depends on a conserved network of structural genes of the melanin synthesis pathway whose expression levels may differ between different body organs [38]. We investigated this hypothesis within two segments: the 2^nd^ thoracic segment (mesonotum) and the 4^th^ abdominal segments (A4), which are developmentally four segments apart. Previous studies in *Drosophila* showed that the degree of genetic correlation of pigmentation decreases with increasing distance between body segments [49],[50]. In agreement with these studies, we did not find a significant correlation between the two segments in the lowland ZI population which is presumably under no directional selection for pigmentation. On the other hand, pigmentation traits correlated between the two segments in the high-altitude Ethiopian population, potentially due to selectively favored alleles that alter multiple pigmentation traits. A recent experimental selection study has shown the presence of correlated response to selection between the thorax and the 2^nd^ abdominal segment [41]. Geographical variation of A4 pigmentation in sub-Saharan Africa and of thoracic trident in Australia were found to be related to differences in *ebony* expression levels [7],[13],[16], but detailed molecular dissection of the regulation of *ebony* expression revealed that different enhancers of the gene affect different body parts [7],[10],[51].

### Adaptive significance of *Drosophila* melanism

Latitudinal or altitudinal clines of abdominal and thoracic pigmentation has previously been found in *D. melanogaster*[13]–[20] and also in other drosophilid species [21]–[25]. In all cases darker phenotypes were encountered at high latitudes or altitudes, but the exact cause of the clinal variation remained elusive. The most invoked hypothesis was the thermal budget or thermal melanism stating that darker flies absorbs better solar radiation in colder environments [32]. This might explain some longitudinal clines too [52]. However, the high surface to volume ratio of these small insects may preclude them from maintaining a higher body temperature than their surroundings [53]. There is little experimental evidence that dark flies are warmer [54], and no experiment to our knowledge has shown a higher fitness of dark morphs in colder environments. The second most cited hypothesis is desiccation resistance. In India, variation in desiccation resistance parallels latitudinal and altitudinal clines of pigmentation [55], and there is experimental evidence that dark morphs are more resistant to desiccation than light morphs due to cuticular thickness [19]. However, the altitudinal cline of pigmentation in sub-Saharan *D. yakuba* was not associated with desiccation resistance [25].

We conducted intensive correlation studies between clinal variation in pigmentation and different environmental factors and found that UV radiation was the strongest predictor of pigmentation traits especially for thoracic pigmentation (MPL). For abdominal pigmentation (A4 background), UV was also the strongest explanatory factor when factors were analyzed separately, but combinations of other factors such as temperature and population history might also be relevant. We did not find any relevance to atmospheric pressure or relative humidity, the two factors which may be the most explanatory for a desiccation-resistance hypothesis. In endotherms, pigmentation tends to increase with decreasing latitudes, the so-called Gloger's rule [5] (cf. [56] for an example in humans). In *Drosophila*, the opposite was always encountered, *i.e.* a positive relationship between pigmentation and latitude, in Europe, India and Australia [14]–[16],[18]. However, David et al. [14] noted that latitudinal clines for thoracic pigmentation were only found in the temperate region encompassing North Africa and Europe (above 30°), whereas pigmentation in India also correlated with altitude which covaries with latitude [15]. Pool and Aquadro [13] found a negative latitudinal cline in sub-Saharan Africa, but the cline was not significant when they corrected for altitudinal effect. In our study, the negative latitudinal cline persisted even after correcting for altitudes. This may be due to our inclusion of high-altitude populations from both higher and lower latitudes. Our study thus provides the first evidence of a negative latitudinal cline of abdominal and thoracic pigmentation in sub-Saharan Africa in agreement with Gloger's rule but for ectotherms.

Considering altitudinal clines of pigmentation, it has long been suggested that the “ecological importance of melanism at high altitudes is closely bound up with the fact that dense pigments serve as effective protection against the injurious effects of the intense ultra-violet” (p. 53, [57]). Scott [58] was “struck by the unicolorous black hue of the body” of some snails and one chloropid fly in high altitudes in Ethiopia, and we report here a uniquely dark morph of *D. melanogaster* from Ethiopia as well. Pool and Aquadro [13] demonstrated the presence of altitudinal clines of abdominal pigmentation in sub-Saharan Africa. Curiously, there is no obvious altitude-pigmentation cline in *D. simulans* (JEP, personal observation), even though this species is thought to spend more time outdoors than its close relative [59], and exists at high altitudes in Africa (e.g., Dodola, Ethiopia, where pigmentation is essentially a diagnostic trait between *D. simulans* and *D. melanogaster*).

Laboratory exposure of *D. melanogaster* to elevated UV radiation has typically shown wild type flies to be more resistant than light or dark pigmentation mutant strains [34]–[36], but flies experimentally fed melanin were found to be more resistant to irradiation [60]. Recently, Matute and Harris [25] reported the association between an altitudinal cline of pigmentation in *Drosophila yakuba* and UV resistance on two islands near western Africa. Surprisingly, the authors found that *lightly* pigmented strains had the longest survival after exposure to high levels of UV radiation. In contrast, the correlations we observe are consistent with a protective effect of dark pigmentation against UV radiation. These species could conceivably differ in the relationship between pigmentation and UV protection. Another possibility is that resistance to elevated UV doses in the laboratory may differ in important ways from UV resistance under natural conditions (*e.g.* due to the release of heat by melanin exposed to high levels of UV in the lab). Future UV resistance experiments with light and dark populations of *D. melanogaster* should be conducted to explain clinal variation in sub-Saharan Africa.

Our analyses were conducted on flies raised under similar laboratory conditions, and hence investigate genetic differences between populations. However, the phenotypes that occur in nature may be modulated by phenotypic plasticity [61], such as the documented influence of developmental temperature on *D. melanogaster* pigmentation [22],[49],[62]–[68], which could also differ between populations. Hence, it will also be worthwhile to study the influence of temperature and other environmental factors on the pigmentation of *D. melanogaster* from Ethiopia and elsewhere.

## Conclusion

Ethiopian populations of *D. melanogaster* display uniquely melanic phenotypes not observed in other worldwide populations. Dark abdominal and thoracic pigmentation in Ethiopian flies appears to have a partly shared genetic basis. Resistance to ultraviolet radiation provides a promising hypothesis for geographic patterns of pigmentation among African populations.

## Competing interests

The authors declare that they have no competing interests.

## Authors’ contributions

HB carried out the phenotypic scoring and drafted the manuscript. AY performed the statistical analyses and drafted the manuscript. EJJ maintained the fly stocks and took digital photos. JEP conceived the study, and participated in its design and coordination and helped to draft the manuscript. All authors read and approved the final manuscript.
